# 

**Published:** 1881-04

**Authors:** 


### CONTENTS:

*ORIGINAL.* Page.
Liberty of Medical Opinion and Action, - Editorial, - - - - - - - 123
The Organon: Paragraph Third, - - Ad. Lippe, M. D., Phila. - - - - - 131
The *Observer* Introductory, - - - "International." - - - - - - 135
An Open Letter from, - - - - - T. F. Pomeroy, M. D., Jersey City, - - 137
Fatal Errors, - - - - - - - - Ad. Lippe, M.D., Phila. - - - - - 139
Haemorrhoids and their Treatment, - - Dr. A. Charge, Paris, - - - - - 141
*CLINICAL CASES:*
*Phosphorus*—Sexual Weakness; *Arsenic*—Piles; *Sulphur*—post-partum Melancholia;
*Sulphur*—Piles; *Phos.*—Deafness, E. W. Berridge, M. D. - - - - - 146
*Baptisia*—Typhoid; *Aloes*—Constipation, E. B. Nash, M. D. - - - - - - 152
Periscope, - - - - - - - - - - - - - - - - - - - - - - - - - 154
*BOOK NOTICES & REVIEWS:*
Catarrhal Diseases, by Dr. Brigman. A. L. Chatterton Publishing Co. - - - 164
World's Hom. Convention of 1876, by Dr. J. C. Guernsey, A.M., Inst. of Homœopathy, 164
International Hahnemann Association. - - - - - - - - - 164
*ITEMS:* - - - - - - - - - - - - - - - - - - - - - 165

